# Plasmonic cavity-apertures as dynamic pixels for the simultaneous control of colour and intensity

**DOI:** 10.1038/ncomms8133

**Published:** 2015-05-20

**Authors:** Hansik Yun, Seung-Yeol Lee, Keehoon Hong, Jiwoon Yeom, Byoungho Lee

**Affiliations:** 1 National Creative Research Center for Active Plasmonics Application Systems, Inter-University Semiconductor Research Center and School of Electrical Engineering, Seoul National University, Seoul 151-744, Republic of Korea

## Abstract

Despite steady technological progress, displays are still subject to inherent limitations in resolution improvement and pixel miniaturization because a series of colours is generally expressed by a combination of at least three primary colour pixels. Here we propose a structure comprising a metal cavity and a nanoaperture, which we refer to as a cavity-aperture, to simultaneously control the colour and intensity of transmitted light in a single pixel. The metal cavity constructs plasmonic standing waves to organize the spatial distribution of amplitudes according to wavelength, and the nanoaperture permits light with a specific wavelength and amplitude to pass through it, depending on the nanoaperature's relative position in the cavity and the polarization state of the incident light. Therefore, the cavity-aperture has the potential to function as a dynamic colour pixel. This design method may be helpful in developing various photonic devices, such as micro-imaging systems and multiplexed sensors.

## Abstract (web summary)

The increased miniaturization of optical technologies necessitates ever smaller pixels, but most routes fix pixel colours upon fabrication. Yun *et al*. show that combined plasmonic cavities and nanoapertures offer a route to nanoscale pixels whose colour and intensity can be dynamically tuned via polarization.

## Graphical abstract

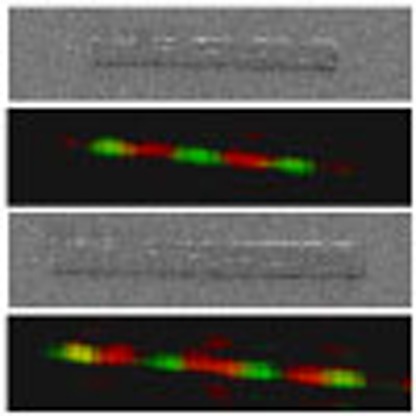

Modern society requires advanced display systems with high resolution and rapid response time in a ubiquitous environment due to the explosive increase of information. With this recent trend, the miniaturization of display pixels provides a route to high-resolution portable display systems. Many research groups in the display industry have attempted to utilize fast dynamics in the compact dimensions of surface plasmons (SPs), which are electromagnetic waves generated by the collective oscillations of photons and electrons at a metal–dielectric interface^1^. In particular, because the resonance wavelengths of SPs have a strong dependence on the geometry of the metallic nanostructures, the property has been applied to various plasmonic colour filters. For example, the size and shape of metal nanoparticles determine their SP resonance wavelength^2^,^3^, the periodicity of a nanohole array on a metal film induces passbands of extraordinary optical transmission^4^,^5^,^6^, and the distance between the input and output of a metal–insulator–metal waveguide influences the output coupling of the propagating SPs^7^,^8^.

Although the geometry dependence of SPs has advantages in selecting their resonance wavelengths, this dependence makes it difficult to tune the SPs' resonance after the nanostructures are fabricated. Although some research groups have reported various methods for changing a colour or image using the polarization dependence of SPs^9^,^10^, such methods have limitations in terms of expressing dynamic full-colour images because those changes are limited to switching between two static colours or images. Historically, conventional colour pixels have also shown such limitations in most commercial displays because the dyes or emitting materials used in the pixels determine their colours. Three different sub-pixels of red (R), green (G) and blue (B) must be used to adequately express specific colours. As a result, the minimum size of a unit pixel is increased by at least three times, which impedes the enhancement of the spatial resolution of a display panel.

To overcome the limitations associated with resolution by the invariant colour characteristics of conventional colour pixels, we propose a novel system of a dynamic colour pixel that is composed of a metal cavity and a nanoaperture. Because the metal cavity constructs plasmonic standing waves to organize the spatial distributions of amplitudes according to wavelength, we can extract light with a specific wavelength and amplitude through the nanoaperture by controlling its relative position in the cavity and the polarization of the incident light. Electromagnetic waves efficiently pass through the nanoscale aperture without deforming the standing wave in the cavity due to the plasmonic resonance depending on the aperture perimeter^11^,^12^,^13^. In this study, we refer to the combined structure as a ‘cavity-aperture' and investigate the fundamental properties of the cavity-aperture by numerical simulations and experiments. We also verify that the proposed structure has the potential to function as a dynamic colour pixel by demonstrating the modulation of three primary colours and their intensities on an array of multiplexed cavity-apertures depending on the polarization state of the incident light.

## Results

### Concept of a cavity-aperture

Electromagnetic waves generally form standing waves in a cavity when their wavelengths satisfy the Fabry–Perot (FP) resonance conditions of the cavity. If we monitor the field distribution inside the cavity at FP resonance, the field amplitudes are minimized at the nodes of the standing waves and are maximized at the antinodes. The field profiles are continuous between the nodes and antinodes. Hence, an arbitrary field amplitude from zero (at nodes) to the maximum value (at antinodes) of standing waves can be extracted if we access the field value at a specific point in the cavity. We introduce a plasmonic nanoaperture to efficiently extract the electromagnetic field from the standing waves in the cavity and to minimize the deformation of the standing wave. SPs excited at the nanohole aperture on a metal film give rise to a strong transmission enhancement, whereas free space light cannot propagate through an isolated subwavelength hole, and the transmission is typically weak^11^,^12^,^13^. In other words, the nanoaperture can act as a point-like source to selectively re-emit the field at a specific position of SP standing waves organized in the cavity.

**Figure 1:**
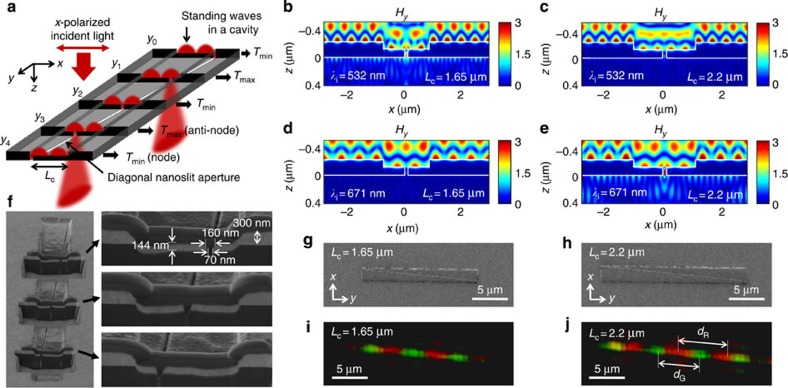
Diagonal nanoslit type of cavity-apertures. (**a**) Schematic diagram of the cavity-aperture. (**b**–**e**) Calculated *H*_*y*_-field profiles of silver cavity-apertures with 1.65 and 2.2 μm cavity lengths for 532 and 671 nm incident light. (**f**) SEM images of 52° tilted cross-sections of the cavity-aperture. The magnified images indicate the structural dimensions of the cavity-aperture and the relative positions of the nanoaperture in the cavity. (**g**,**h**) SEM images of the top views of the cavity-apertures. (**i**,**j**) OM transmittance images of the cavity-apertures.

First, we conceptually outline a structure of dynamic colour pixels with a metal cavity and nanoaperture to systematically select a colour and intensity in a unit pixel. The metal cavity spatially distributes different amplitudes in the cavity according to wavelength, and the nanoaperture extracts a light with a specific wavelength and amplitude from the SP standing waves in the cavity. A cavity-aperture is schematically presented in Fig. 1a and is used to investigate the wavelengths and amplitudes of fields transmitted through it with respect to the relative position of an aperture in a cavity. A nanoslit aperture is engraved diagonally on a rectangular cavity with lengths of *L*_c_ and 10*L*_c_ along the *x*- and *y*-axes, respectively. Because the position of the nanoaperture changes continuously from 0 to *L*_c_ along the *x*-axis in cross-sections of the cavity on *xz*-planes with increasing *y*-value, the diagonal nanoslit is an appropriate structure to use for the continuous observation of the transmittance characteristics, depending on the position of the nanoaperture in the cavity with a fixed *L*_c_. For example, the *x*-polarized light that normally illuminates the cavity-aperture would pass through the slits of the cross-cut cavities at *y*_1_ and *y*_3_ with a maximum intensity due to the overlap of the antinodes and the nanoslit, but it would pass through the slits at *y*_0_, *y*_2_ and *y*_4_ with a minimum intensity due to the overlap of the nodes and the nanoslit. In addition, in the case of two SP standing waves with different wavelengths, if we determine the cavity length where the nodes of one wavelength overlap with the antinodes of the other wavelength, the respective positions for their maximum transmittances would alternate along the diagonal nanoslit.

### Optical characterization of a cavity-aperture

To confirm the construction of SP standing waves in a metal cavity, we calculate an *H*_*y*_-field profile for a silver (Ag) cavity-aperture using the two-dimensional rigorous coupled wave analysis (RCWA) method^14^,^15^ when *x*-polarized light illuminates the cavity-aperture in the *z*-direction. Although the fields are affected by all structural parameters, we fix the parameters other than the cavity length (*L*_c_) to focus on the effect of the cavity length because *L*_c_ has a direct influence on the construction of the SP standing waves. The metal thickness (*T*_m_), cavity depth (*D*_c_) and aperture width (*W*_a_) are fixed at 300, 150 and 50 nm, respectively, and the aperture is located at the centre of the cavity. As shown in Fig. 1b,c, the *H*_*y*_-field profiles of the cavity-aperture in the *xz*-plane show that SP standing waves with odd and even periods are formed when incident light with a wavelength (*λ*_i_=532 nm) illuminates two different cavities (*L*_c_=1.65 and 2.2 μm), respectively. The field penetrating through the 50-nm-width nanoaperture does not destroy the overall shape of the standing waves. The transmittance of the cavity-aperture reaches a maximum (or minimum) when the location of the aperture matches that of the antinode (or node), as shown in the case of odd (or even) periods. Let us expand the case by simultaneously illuminating two different incident wavelengths of 532 and 671 nm in a cavity, as shown in Fig. 1b–e. When *L*_c_ is 1.65 μm, the 532 nm light forms a standing wave with an odd number of periods, whereas an even number of periods is formed for the 671 nm light. However, these aspects are reversed when a cavity length of 2.2 μm is used. Therefore, 532 and 671 nm wavelengths can be successfully transmitted through the nanoapertures in cavities with lengths of 1.65 and 2.2 μm, respectively, but they are almost completely blocked in cavity-apertures with lengths of 2.2 and 1.65 μm, respectively.

We fabricate a diagonal slit in a rectangular cavity on an Ag layer through e-beam evaporation and focused ion beam (FIB) milling to measure the optical characteristics of the cavity-aperture in the experiments described below. Figure 1f shows field emission scanning electron microscope (SEM) images of three cross-sections of the 52° tilted cavity-aperture. The structural dimensions of the sample are consistent with those of the simulated structure, and the diagonal nanoslit aperture penetrates the centre and the off-centre, as shown in three magnified images of the cross-sections. Figure 1g,h shows top-view images of cavity-apertures with cavity lengths of 1.65 and 2.2 μm, and Fig. 1i,j is the transmittance images of the cavity-aperture, as measured by an optical microscope (OM) when *x*-polarized green (532 nm) and red (671 nm) lasers simultaneously illuminate the sample. In the case of *L*_c_=1.65 μm (Fig. 1i), the green laser is transmitted at the centre of the diagonal slit with a maximum intensity, and the red and green lasers are alternately and periodically transmitted along the slit. The positions of the antinodes of the 532 and 671 nm wavelengths in Fig. 1b,d are in accord with those of the green and red colours shown in Fig. 1i. In the case of *L*_c_=2.2 μm (Fig. 1j), the red colour is observed at the centre of the nanoslit aperture because the antinode of the standing wave produced by the 671 nm laser is at the centre of the slit, as opposed to the case presented in Fig. 1i. The number and position of the two colours on the diagonal slit aperture coincide to a significant extent with those of the calculated *H*_*y*_-field profiles. The wavelengths of the SP standing waves for two incident lights (532 and 671 nm) are measured as 519 and 657 nm in Fig. 1j because the distances (*d*_G_ and *d*_R_) between the maximum transmittance points of the R and G colours are 5.2 and 6.6 μm, respectively. These values are similar to the theoretical wavelengths of SPs (505.4 and 652.7 nm).

**Figure 2:**
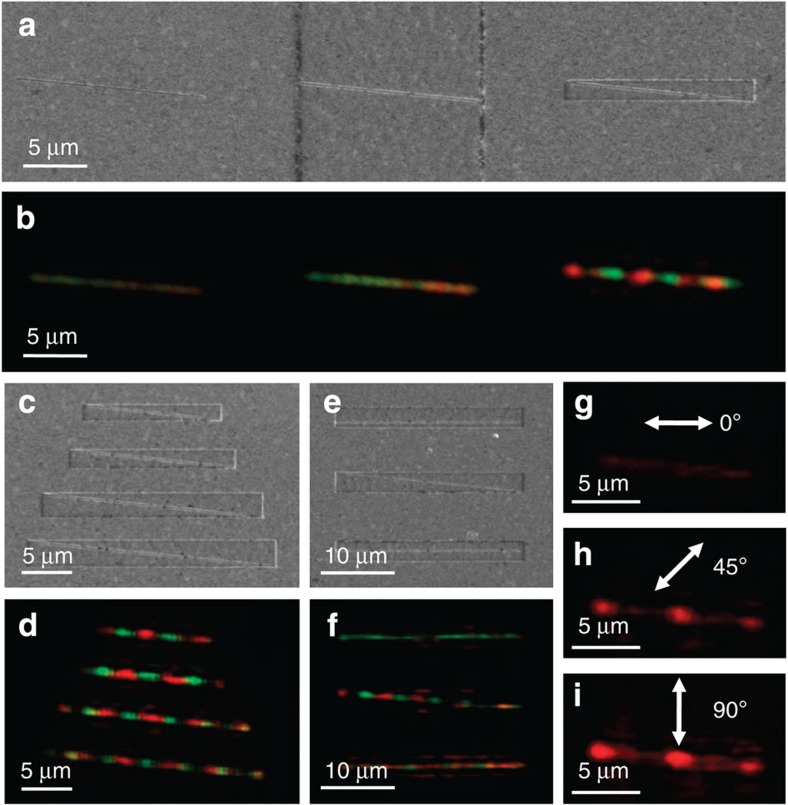
Effects of the structural parameters and optical polarizations on the transmittance properties of cavity-apertures. SEM and OM images of the effects of (**a**,**b**) cavities (the left slit: no cavity, the middle slit: 100-μm-long cavity, the right slit: 1.3-μm-short cavity), (**c**,**d**) cavity lengths (1.3, 1.65, 2.2, 2.5 μm from the top) and (**e**,**f**) nanoslit positions in cavity-apertures (the top slit: off-centred, the middle slit: diagonal, the bottom slit: on-centred), respectively. (**g**–**i**) OM images of the effects of the polarization state of a 671-nm incident laser.

To further investigate the effect of the cavity, we measure the transmittances of three diagonal slit apertures without a cavity (the left slit), with a 100-μm-long cavity (the middle slit), and with a 1.3-μm-short cavity (the right slit) in one sample, as shown in Fig. 2a,b. The alternate transmission of two colours only appears along the slit in the cavity-aperture with the cavity length of 1.3 μm (the right slit), whereas it is not observed in the other slit apertures (the left and the middle slits). This serves to verify that the antinodes of the SP standing waves are strongly generated only by FP resonances in the short cavity. On the contrary, it is impossible to satisfy FP resonances in the case of the slit aperture on the flat Ag layer, and FP resonances also do not appear in the case of the long cavity due to a large amount of energy loss that occurs during the back and forth propagation of SPs on the metal surface. To evaluate the energy efficiency of the cavity-aperture, we analyse the energies of light converted to SPs, light lost due to dissipation, and light transmitted in the cavity-aperture using the RCWA simulation. Based on the input power illuminating the inner region of the cavity, ∼90.1% of the incident light is converted to SPs in the cavity, ∼50.3% is dissipated in the metal and ∼39.8% of the light is finally transmitted through the cavity-aperture. The amount of light converted to SPs and the transmitted light in the cavity-aperture is approximately six and four times higher than in a conventional aperture without a cavity, which are 14.8 and 10.4%, respectively. It is also experimentally confirmed that the presence of a cavity enhances the transmittance of the nanoaperture. The transmittance of the cavity-aperture (the right slit) in Fig. 2b is approximately five times higher than that of the typical aperture (the left slit) in Fig. 2b. Therefore, the cavity plays a significant role in organizing the spatial distributions of amplitudes according to wavelengths and in enhancing the transmittance of the aperture within it.

It is important to ascertain whether the transmittance properties in a cavity-aperture can be controlled in the case of an application for dynamic display pixels. Figure 2c,d demonstrates the influence of the cavity length on the number of bright transmission spots. When the dimensions of the rectangular cavities are gradually enlarged with the ratio of width and length being held constant, the number of bright transmission spots increases. This can be attributed to the fact that the number of antinodes of the standing waves increases with increasing *L*_c_ value. In addition, changing the position of the slit aperture in the cavity and the polarization angle of the incident lasers serves to confirm that the colour and intensity of the transmitted light in a cavity-aperture can be modulated, as shown in Fig. 2e–i. When the slit is placed in parallel with the cavity where its lateral location is off- and on-centre with respect to the cavity (the top and the bottom slits in Fig. 2e), only one of the laser sources passes through the cavity-aperture (the top and the bottom slits in Fig. 2f), in contrast to the diagonal slit aperture (the middle slit in Fig. 2e,f). As the polarization angle of the 671-nm laser is rotated from parallel to perpendicular polarization to the nanoslit, such as 0°, 45° and 90°, the intensity of the transmittance changes from a minimum to a maximum because only the perpendicular component of the incident light to the longer axis of the cavity-aperture contributes to FP resonances, as shown in Fig. 2g–i. Therefore, the colour and intensity of the transmitted light can be modulated in the cavity-aperture by changing the relative position of the antinodes and the nanoaperture, and the polarization state of the incident light.

### Application of multiplexed cavity-aperture to dynamic pixel

On the basis of controllable transmittance properties of the diagonal slit aperture in the rectangular cavity, it is possible to determine the three cavity lengths required to clearly express the three primary colours of R, G and B with a maximum intensity at the centre of the cavity-aperture and to design a dynamic display pixel by superimposing three types of cavity-apertures with different rotational angles to modulate the colour and intensity controlled by optical polarization.

**Figure 3:**
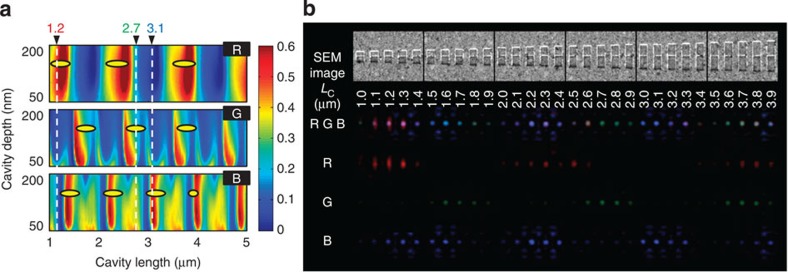
RGB transmittance map of the cavity-apertures. (**a**) Simulation map. (**b**) SEM and OM images for the experimental map. The experimental results are marked with yellow ellipses on the simulation map to permit convenient comparisons. The cavity lengths needed to pass each of the RGB colours through the cavity-apertures with a maximum intensity are 1.2, 2.7 and 3.1 μm, respectively. They are marked with white dotted lines on the simulation map.

For cavity-apertures illuminated by incident light with wavelengths of 671 nm (R), 532 nm (G) and 473 nm (B), transmittance graphs depending on the cavity length and depth (RGB transmittance map) are calculated for a range of cavity lengths (1–5 μm) and depths (50–200 nm), as shown in Fig. 3a. The maximum and minimum transmittances alternate periodically because the antinodes and nodes of the standing wave are located periodically and alternately on the centred nanoslit aperture in the cavity as the cavity length changes. The period is reduced with decreasing wavelength because the period of the standing wave is dependent on the incident wavelength. An RGB transmittance map of the cavity-aperture structures is also obtained experimentally. A sample for mapping can be prepared as a series of centred nanoslit cavity-apertures with various cavity lengths with differences of 100 nm from 1 to 3.9 μm, as shown in the upper SEM images of Fig. 3b. We acquire the experimental map by simultaneously or separately illuminating the sample with R, G and B lasers, as shown in Fig. 3b. The results indicate that the three colours appear with different periods in rows, ‘R', ‘G' and ‘B'. In addition, various colours can be successfully expressed in row ‘RGB' through the mixing of the three primary colours. The yellow ellipses in Fig. 3a indicate the positions where each primary colour is located on the experimental map. These results confirm that the experimental map is in significant agreement with the theoretical results.

**Figure 4:**
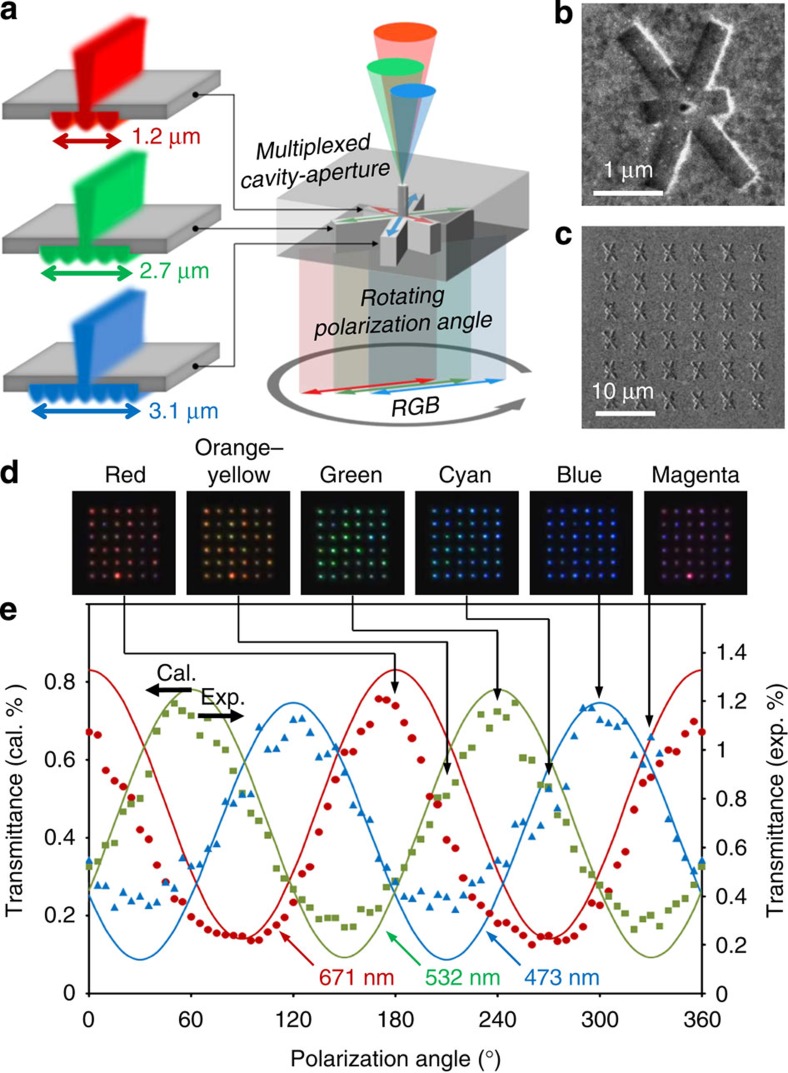
Application of cavity-apertures to dynamic colour pixels. (**a**) Schematic diagram of the multiplexed cavity-aperture. (**b**,**c**) SEM images of a unit pixel and a 6 × 6 array of the multiplexed cavity-apertures. The aperture size is 50 nm. (**d**) OM transmittance images of the multiplexed cavity-aperture array. Red, orange–yellow, green, cyan, blue and magenta colours are expressed. (**e**) Graph of the absolute transmittance versus polarization angle for the cavity-aperture array. The simulation and experimental data of the absolute transmittance are marked with the solid curves and the data points, respectively. The OM images in **d** are matched to the respective polarization states using the solid lines and the arrows. Cal., calculated; Exp., experimental.

It is possible to determine three specific cavity lengths that produce high transmittance for one primary colour but have low transmittances for the other primary colours because the period and position of the maximum transmittance values for each colour are all different in the RGB transmittance map. Based on the RGB transmittance map shown in Fig. 3a, cavity lengths of 1.2, 2.7 and 3.1 μm (white dotted lines of Fig. 3a) are selected for single primary colour transmission for R, G and B colours, respectively. Next, we rotate and overlap these three cavities with different lengths by 60° to minimize the interference among them and create a nanohole aperture at the centre that is common with respect to the three cavities, as shown in Fig. 4a. When the polarization of the incident light is parallel with one of three cavities and the nanohole matches the antinode of the standing wave, the multiplexed cavity-aperture can act as a unit pixel to selectively pass only one of the RGB colours. Figure 4b,c shows SEM images of the unit pixel and 6 × 6 pixel array of the multiplexed cavity-apertures. When the polarization of the RGB lasers is rotated from 0° to 180°, both the colour and intensity of the transmitted RGB lasers change. Owing to combinations of the colour and intensity of the RGB sources, the array of the multiplexed cavity-apertures expresses red, orange–yellow, green, cyan, blue and magenta colours, in order, as shown in Fig. 4d. The continuous change is presented in Supplementary Movie 1. It is possible to prepare two types of graphs of polarization state versus absolute transmittance quantitatively based on the observed OM images and the simulation results, as shown in Fig. 4e. The absolute transmittance is defined as the power ratio of the total transmitted light to the total incident light in a rectangular area covering one multiplexed cavity-aperture. The maximum transmittances are approximately 1%, which are 0.8% in the simulation and 1.2% in the experiment. The difference between the experimental and simulation values is estimated to originate from errors associated with the fabrication of aperture sizes in the FIB milling processes, such as nonhomogeneous milling and edge blurring of the nanoapertures. Despite the low transmittance, one of three primary colours has a maximum transmittance at different angles of 60° from the polarization angles where the other two colours have relatively high transmittances, and the intensity of each colour follows a sinusoidal curve. This result indicates that the multiplexed cavity-aperture successfully guides the antinodes of three different standing waves to the centred hole aperture. Therefore, the proposed multiplexed cavity-aperture can express various colours through the three primary colours, and has the potential for creating dynamic pixels for use in a display.

## Discussion

We demonstrated the fundamental properties and potential of a cavity-aperture to function as a dynamic colour pixel in displays. The cavity-apertures can be used to generate the full range of the colour spectrum depending on the polarization state of the incident light, in contrast to the static or discrete colours that are produced by conventional colour pixels. This simultaneous controllability of colour and intensity, which is distinct from the geometry-dependent resonance properties of typical plasmonic structures, is acquired through a new approach, in which two fundamental characteristics of the metal cavity and the nanoaperture in nanophotonics are combined.

Further follow-up studies are needed to practically apply the cavity-aperture to a pixel structure of a display panel. First, the low transmittance of the multiplexed cavity-apertures is insufficient for displays, as the transmittance of the most widely used display pixels is generally between 5 and 10% (refs 16, 17). Therefore, the nanostructure should be optimized to improve the transmittance. With regard to apertures, C-, H- and bowtie-shaped apertures have significantly higher transmission efficiencies in comparison with conventional nanohole apertures^18^,^19^,^20^,^21^ because the plasmonic resonance depending on the aperture perimeter induces a strong charge accumulation at the sharp edges of the unconventional nanoaperture^22^,^23^. Because this principle can also be applied to a nanoaperture in a cavity, the adoption of the novel design will provide opportunities to address the low transmission problem. With regard to the three-cavity part, the dimensional optimization by tuning the wavelengths of the laser sources and the RGB transmittance map will minimize the pixel size and increase the transmission density. The minimal cavity size based on the RGB transmittance map in Fig. 3a can be reduced to less than 2 μm, which is sufficiently small in comparison with the pixel sizes of commercial TV and mobile phone displays, which are approximately 250 and 100 μm (refs 24, 25). In addition to these types of structural optimizations of the cavity-aperture, high-density packing of the cavity-apertures, such as a honeycomb lattice, would also help to improve their transmittance and resolution. Individual polarization states corresponding to each cavity-aperture should be independently controlled for the dynamic pixels in their array to be realized. Many research groups have investigated the use of pixelated polarizers^26^,^27^,^28^,^29^ and the integration of colour filters and polarizers^30^,^31^ based on the micro- and nano-patterning of liquid crystal polymers or nanowires. Similar cases in the field of plasmonic colour filters^6^,^32^ will be helpful in synchronizing connections between cavity-apertures and electro-optic devices for displaying a dynamic image.

The cavity-aperture has significant advantages in terms of size, resolution and response time because it is a type of plasmonic structures, despite having a low transmittance. These properties make the cavity-aperture more appropriate for application in advanced displays than general displays. Whereas advanced displays, such as three-dimensional and holographic displays, require high-resolution and high-pixel density^33^,^34^, the typical pixels of liquid crystal displays have limitations in scaling down because crosstalk increases between the voltages of neighbouring pixels at dimensions of a few micrometres^35^,^36^. The proposed method of the cavity-aperture can be helpful in scaling pixels down and increasing pixel density due to the advantage of expressing a series of colours with a single pixel instead of three primary colour pixels. Considering an example of the application in head-mounted displays or micro-displays, the laser sources can be relayed to the highly packed cavity-apertures via optical fibres or waveguides^37^,^38^,^39^, and such high-resolution images would satisfy the resolution of human eyes even after the field of view is expanded over a short distance or in a small panel^40^,^41^. Therefore, we expect that the cavity-aperture can be applied as a novel type of display pixel after further development. In addition, the approach and methodology reported in this study can be utilized in innovating various photonic devices, such as micro-imaging systems and multiplexed sensors.

## Methods

### Numerical simulations

For the calculation of the electromagnetic fields and transmittance of the proposed structure, our in-house constructed RCWA tool was used^14^,^15^. The numerical method first divides the overall geometry as an array of layers and calculates the Fourier spectrum of each layer. The resulting eigenmodes that can exist in those layers are then obtained. Finally, the coupling coefficients from the incident wave to the eigenmodes in each layer are calculated using the extended transfer matrix method. By summing the coupling coefficients for all of the eigenmodes existing in each layer, it is possible to obtain *H*_*y*_-field profiles and the transmittance of various cavity-apertures for a specific wavelength.

### Sample fabrication and optical measurement

A thin silver layer with a 150-nm thickness was deposited on a clean glass substrate using an e-beam evaporator (KVE-3004, Korea Vacuum Tech.). Cavities with a depth of 200 nm were milled with a FIB (Quanta 200 3D, FEI). After deposition of a 150-nm-thick silver layer on the cavity-patterned sample, nano-slit or nano-hole apertures were milled at the cavities with the FIB. Top and cross-section views of the cavity-apertures were measured by a field emission SEM (JSM 6700F, JEOL). Red (671 nm), green (532 nm) and blue (473 nm) lasers were aligned to one optical path by using dichroic mirrors, and the cavity-apertures were illuminated normally through an adjustable polarizer. The opposite surfaces of the samples were observed using the modified OM setup, which has a charge-coupled device (XCD-SX90CR, Sony) and an objective lens with a magnitude of 100 and a numerical aperture of 0.8 (LMPlanFLN, OLYMPUS Corp.).

## Additional information

**How to cite this article:** Yun, H. *et al*. Plasmonic cavity-apertures as dynamic pixels for the simultaneous control of colour and intensity. *Nat. Commun.* 6:7133 doi: 10.1038/ncomms8133 (2015).

## Supplementary Material

Supplementary Movie 1 The transmitted colour of the 6 × 6 array of the multiplexed cavity-apertures in Figure 4c continuously changes from red to magenta via orange-yellow, green, cyan, and blue, as the polarization angle of the incident light increases from 0° to 180°. The incident light sources are red (671 nm), green (532 nm), and blue (473 nm) lasers.
